# Outcomes of Liver Resections after Liver Transplantation at a High-Volume Hepatobiliary Center

**DOI:** 10.3390/jcm9113685

**Published:** 2020-11-17

**Authors:** Julian M. O. Pohl, Nathanael Raschzok, Dennis Eurich, Michael Pflüger, Leke Wiering, Assal Daneshgar, Tomasz Dziodzio, Maximilian Jara, Brigitta Globke, Igor M. Sauer, Matthias Biebl, Georg Lurje, Wenzel Schöning, Moritz Schmelzle, Frank Tacke, Johann Pratschke, Paul V. Ritschl, Robert Öllinger

**Affiliations:** 1 Department of Surgery, Campus Charité Mitte, Campus Virchow-Klinikum, Experimental Surgery, Charité—Universitätsmedizin Berlin, Corporate Member of Freie Universität Berlin, Humboldt-Universität zu Berlin, and Berlin Institute of Health, 13353 Berlin, Germany; julian.pohl@charite.de (J.M.O.P.); nathanael.raschzok@charite.de (N.R.); dennis.eurich@charite.de (D.E.); michael.pflueger@charite.de (M.P.); leke.wiering@charite.de (L.W.); assal.daneshgar@charite.de (A.D.); tomasz.dziodzio@charite.de (T.D.); maximilian.jara@charite.de (M.J.); brigitta.globke@charite.de (B.G.); igor.sauer@charite.de (I.M.S.); matthias.biebl@charite.de (M.B.); georg.lurje@charite.de (G.L.); wenzel.schoening@charite.de (W.S.); moritz.schmelzle@charite.de (M.S.); johann.pratschke@charite.de (J.P.); paul.ritschl@charite.de (P.V.R.); 2 BIH Charité Clinician Scientist Program, Berlin Institute of Health (BIH), 13353 Berlin, Germany; 3 Department of Hepatology and Gastroenterology, Campus Virchow Klinikum and Campus Charité Mitte, Charité University Medicine Berlin, 13353 Berlin, Germany; frank.tacke@charite.de

**Keywords:** liver transplantation, liver resection, hepatocellular carcinoma, graft survival, ischemic type biliary lesions

## Abstract

Although more than one million liver transplantations have been carried out worldwide, the literature on liver resections in transplanted livers is scarce. We herein report a total number of fourteen patients, who underwent liver resection after liver transplantation (LT) between September 2004 and 2017. Hepatocellular carcinomas and biliary tree pathologies were the predominant indications for liver resection (n = 5 each); other indications were abscesses (n = 2), post-transplant lymphoproliferative disease (n = 1) and one benign tumor. Liver resection was performed at a median of 120 months (interquartile range (IQR): 56.5–199.25) after LT with a preoperative Model for End-Stage Liver Disease (MELD) score of 11 (IQR: 6.75–21). Severe complications greater than Clavien–Dindo Grade III occurred in 5 out of 14 patients (36%). We compared liver resection patients, who had a treatment option of retransplantation (ReLT), with actual ReLTs (excluding early graft failure or rejection, n = 44). Bearing in mind that late ReLT was carried out at a median of 117 months after first transplantation and a median of MELD of 32 (IQR: 17.5–37); three-year survival following liver resection after LT was similar to late ReLT (50.0% vs. 59.1%; *p* = 0.733). Compared to ReLT, liver resection after LT is a rare surgical procedure with significantly shorter hospital (mean 25, IQR: 8.75–49; *p* = 0.034) and ICU stays (mean 2, IQR: 1–8; *p* < 0.001), acceptable complications and survival rates.

## 1. Introduction

Liver transplantation (LT) is the treatment of choice for end-stage liver diseases, e.g., liver cirrhosis, acute or chronic liver failure, acute liver failure, autoimmune diseases, vascular anomalies, and metabolic disorders. Within certain criteria, liver transplantation is also the treatment of choice for (malignant) tumors (mostly hepatocellular carcinomas—HCCs). Recurrence of the latter, but also de novo tumors and localized pathologies of the graft (e.g., biliary strictures or abscesses due to vascular complications) may be indications for liver resection in the graft. However, data on liver resections after LT is scarce [1,2,3,4,5,6,7,8,9,10,11,12,13,14,15,16].

In general, due to the high regenerative capacity of the liver, liver resection in the 21st century has become a safe and well-established surgical procedure for the treatment of benign or malignant primary or secondary liver tumors or other, rare nonmalignant or precancerous pathologies [17]. In cases of the previous LT, liver resection may be complicated due to post-transplant adhesions, reduced liver function due to chronic graft damage and the sequelae of chronic immunosuppression (e.g., renal insufficiency). Therefore, patients undergoing liver resection after LT must be carefully evaluated. The main criteria of feasible liver resection comprise an adequate volume of the predicted liver remnant with sufficient residual liver function and complete resection of the pathology should be achieved. Additionally, the patients’ general health status must allow for this major abdominal surgery. As an alternative, in non-oncologic cases (or selected oncologic cases), liver retransplantation (ReLT) and locally ablative procedures for tumors might be considered. However, compared to primary transplantation, ReLT is associated with significantly lower long-term survival and more complicated postoperative courses [18,19,20,21,22]. In addition, the burden of liver disease might be underrepresented under current allocation policies due to a low Model for End-Stage Liver Disease (MELD) score aggravated by growing organ scarcity [5,8,23]. On the other hand, there is a lack of evidence for the success of locally ablative procedures in transplanted livers [10]. Herein, we present our data on liver resections after LT that were performed between September 2004 and December 2017, which—to our knowledge—is the largest patient cohort on this specific topic in the literature (Table 1, [1,2,3,4,5,6,7,8,9,10,11,12,13,14,15,16]). The primary aim of this study is to demonstrate that liver resection after liver transplantation is a safe alternative to other treatment options such as ReLT.

## 2. Methods

### 2.1. Data Acquisition

We retrospectively screened all liver resections and LTs in adults at the Department of Surgery, Charité—Campus Charité Mitte | Campus Virchow Klinikum, Charité—Universitätsmedizin Berlin between September 2004 and 2017, and cases with liver resections after LT and liver retransplantations were identified. Cases were reviewed and recorded in a clinical database approved by the local ethical board (ethics committee of the Charité; application number EA1/369/16).

### 2.2. Liver Resection and ReLT

All resections after LT were carried out in an open technique according to our institutional standards using intermittent vascular exclusion (if applicable), as well as ultrasonic dissection, ligatures and titanium clips. Postoperative complications were classified according to Clavien–Dindo [24]. No patient received additional locoregional treatment in addition to liver resection in this cohort. For most indications ReLT displays the only therapeutic alternative; hence, ReLT patients served as a control group. Therefore, a subgroup analysis within the resection group has been carried out, excluding patients with a contraindication (e.g., HCC outside Milan criteria, spreading cancer disease, septic shock) for potential ReLT. ReLT was carried out according to our local standard with grafts from deceased donors only.

For better comparability among patients who underwent ReLT, we defined a subgroup (late ReLT) excluding patients:

- with ReLT earlier than twelve months after primary LT,
- high urgency transplantation.

### 2.3. Immunosuppression

Immunosuppressive therapy after LT comprised tacrolimus (target trough level in postoperative weeks 1 to 4, 6 to 10 ng/mL; weeks 5 to 8, 5 to 8 ng/mL) and prednisone (initial dose of 40 mg/day). Prednisone was tapered until the postoperative week 6 in patients without autoimmune disease or left at baseline levels (5 mg/day). Immunosuppression was converted to cyclosporine in case of tacrolimus side effects. Mycophenolate mofetil (MMF) was added in selected cases or during follow-up (e.g., renal insufficiency or autoimmune disease). According to the danger hypothesis [25] and our institutional standard for liver resections, patients received 250 mg methylprednisolone shortly before liver resection. In cases of tumor diagnosis, everolimus, due to its anti-tumor properties, [26] is being introduced in patients with malignancies. Immunosuppression in ReLT patients consisted of the same immunosuppression regimen, in selected cases, the dosage was individually increased. The immunosuppression of the liver resected individuals at time of resection can be seen in Table 2.

### 2.4. Statistics

Survival was calculated by Kaplan–Meier analysis. The log-rank test was used to compare survival between groups. The Mann–Whitney U-test was used for comparison of non-parametric data. *p*-values < 0.05 were considered statistically significant. Means with standard deviations (SD) and medians with interquartile ranges (IQR) have been used depending on the distribution of the data, which was checked by visual comparison of histogram plots.

Statistical analyses and graphs were performed using SPSS Version 26 for macOS (IBM, Armonk, NY, USA).

## 3. Results

### 3.1. Liver Resections

Between September 2004 and 2017, 4100 liver resections have been carried out at our center, amongst them, 14 in patients having previously undergone LT (0.34%). Median follow-up was 25 months (IQR: 4.75–55.75). Four patients were female (28.6%). Mean age at time of liver resection was 57.8 (SD ± 10.85) years with a median MELD score of 11 (IQR: 6.75–21; Table 3) and the procedure was performed 120.5 months (IQR: 56.5–199.25) after transplantation. The median postoperative hospital stay was 25 days (IQR: 8.75–49) with a median of 2 days (IQR: 1–8) in the intensive care unit (ICU). Indications for liver resection were HCCs (n = 5; 35.71%; de novo, n = 2; recurrence, n = 3), biliary tree pathologies (n = 5; 35.71%), liver abscesses (n = 2; 14.28%), exclusively intrahepatic post-transplant lymphoproliferative disorder (PTLD) (n = 1; 7.14%) and one benign hepatic lesion, where initially HCC recurrence was suspected (n = 1, 7.14%). The majority of patients had left hemihepatectomy (n = 6; 42.9%), four patients bi- or tri-segmentectomy (V, VIII; II, III; V, VI; IV, V, VIII; 28.6%) and two patients had a right hemihepatectomy (14.3%), or segmentectomy (IV; VI; 14.3%), respectively (Table 2). Two patients received additional resections after LT prior or beyond the observational period. One patient received segmentectomy of segments II and III in 2001 for HCC recurrence in the liver graft. In 2006, this patient was resected again (segments V and VI) for HCC recurrence and is still alive without any indication for recurrence. A second patient, after liver resection in the patient’s transplanted liver in 2017, had two more local resections in 2018 and 2019 for HCC recurrence.

### 3.2. ReLTs

In the same time period, 175 patients underwent ReLT. Indications were primary non-function of the graft (n = 41; 23.43%), hepatic artery thrombosis (n = 37, 21.14%), ischemic type biliary lesions (ITBL, n = 28, 16.0%), recurrent disease after LT (n = 20, 11.43%), rejection of the graft (n = 14, 8.0%) and other unspecified indications (n = 35, 20.0%). The majority of the patients were male (n = 96; 54.9%). Mean age at ReLT was 49.0 (SD ± 10.7) years, which was significantly lower than in the resection group (*p* = 0.003). ReLT was performed 19.5 days (IQR: 6–2215.75) after first LT, which is significantly earlier than for liver resections after LT (*p* < 0.001). ReLT patients had a preoperative median MELD score of 31 (IQR: 21–36), which was significantly higher compared to the resection group (MELD 11; *p* < 0.001).

ReLT patients were hospitalized postoperatively for a median of 44 (IQR: 25–75) days compared to 25 days (IQR: 8.75–49) in the resection group (*p*= 0.017). In addition, the postoperative ICU stay was significantly longer after ReLT with a median stay of 19 days (IQR: 8–49) compared to 2 days (IQR: 1–8) after liver resection, respectively (*p*= <0.001, Table 3).

When excluding early retransplantations (<1 year and HU), we found that in the remaining “late-ReLT” subgroup (n = 44), indications were ITBL (n = 15, 34.1%), recurrent disease after LT (n = 14, 31.8%), late hepatic artery thrombosis (n = 5, 11.4%) and other unspecified indications (n = 10, 22.7%). The majority of the patients were male (n = 25; 56.8%). Mean patient age was 47.55 years (SD ± 11.76) at the time of late-ReLT, that was carried out at a median of 117.45 months (IQR: 57.9–167.18) after LT, similar to the patients, who received liver resection (117.45 vs. 120.5 months; *p* = 0.778). Late-ReLT patients had significantly higher MELD scores of 32 (vs. 11; *p* < 0.001 vs. resected after LT). Overall median hospital and ICU stay was 40 days (IQR: 24–68.25) and 16 days (IQR: 8–29.75), respectively, and therefore significantly longer in this group than for patients after liver resection (*p* = 0.034 and *p* < 0.001, respectively).

### 3.3. Complications and Survival

After liver resection (post LT), no patient showed signs of acute graft rejection during the perioperative course. In four cases (28.6%), no postoperative complications occurred. Two patients (14.3%) suffered from Clavien–Dindo grade II, three patients (21.4%) from Grade III, and two (14.3%) patients from grade IV complications, both requiring hemodialysis for acute renal failure. Three patients (21.4%) were classified grade V and did not survive the postoperative hospital stay after undergoing liver resection. Indications in those fatal cases were biliary stricture related abscesses of the transplanted liver (n = 2) and metastatic HCC recurrence (n = 1). One patient had to undergo high urgency—ReLT, two days after receiving a right hemihepatectomy for liver abscesses and died shortly after unsuccessful ReLT. The second patient was admitted to our department with recurrent abscesses and sepsis. The finding of an ileus with necrosis of the hepaticojejunostomy was shown intraoperatively and reconstructed. In the further clinical course, the hepaticojejunostomy was again insufficient and re-reconstruction impossible. The patient died on POD 39 due to sepsis and secondary multi-organ failure. The third patient died on POD 46 due to hemorrhagic shock. In the further course, 5 patients died during the follow-up period due to cerebrovascular accident (20 months), sepsis (41 months) and HCC recurrence 6, 7 and 19 months after resection, respectively.

**Figure 1:**
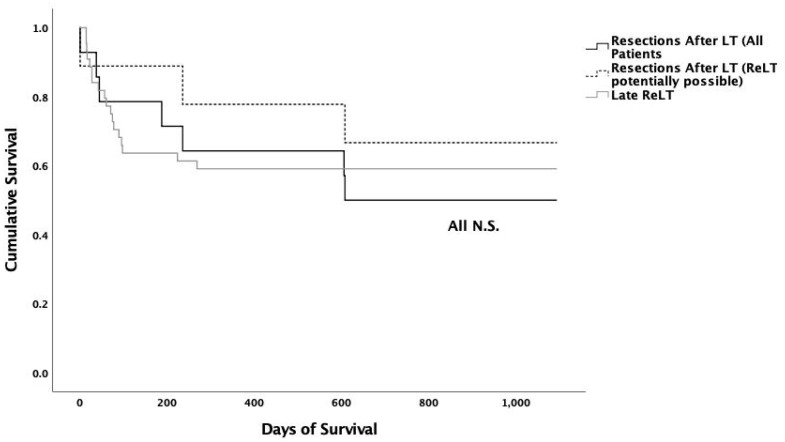
Kaplan–Meier Analysis: Between groups no statistically significant differences for three-year survival could be shown. All liver resection patients vs. late re-transplantation patients three-year survival: 50.0% vs. 59.1% (*p* = 0.733). Liver resection in patients who were potentially retransplantable vs. late re-transplantation patients three-year survival was 66.7% vs. 59.1% (*p* = 0.598).

Three-year survival was 50.0% after liver resection in the liver transplant recipients (Figure 1). Overall three-year survival of ReLT patients was 55.4% (*p* = 0.913 vs. resected, respectively). Patients in the late ReLT group had a three-year survival of 59.1%, respectively (*p* = 0.733 vs. resected, respectively). Liver resected patients classified as potentially transplantable showed a similar three-year survival (66.7%, *p* = 0.598) compared to the patients in the late ReLT subgroup (Figure 1).

## 4. Discussion

Herein, we report the largest single study published so far from a center cohort of patients (n = 14) who received liver resection(s) after LT. Not included in this report are three cases resected at our center after LT that have been previously described [5]. This topic is of special relevance as chronic donor shortages and increasing indications for liver transplantation result in a growing gap of appropriate organs. In the above-described cohort, 9 out of 14 patients were resected who would have otherwise needed a donor organ. In addition, compared to a control cohort of ReLT patients, liver resection resulted in shorter hospital stays, indicating a more harmless treatment. Liver resection is the treatment of choice for distinct pathologies of the liver. Due to technical improvements, complication rates have constantly decreased, and survival rates increased since the 1960s and 1970s, with favorable perioperative and long-term (oncological) outcomes [17,27].

However, data is scarce on liver resections in patients after LT, even though it has been demonstrated to be feasible. The feasibility of liver resections is exemplarily shown in Chari et al., who reported almost full compensation of hepatic tissue in one liver transplant recipient shortly after right hepatectomy [2]. Thus, even major resections seem to be possible after LT. Based on case reports and small case series, outcomes of liver resections after LT, with respect to survival, can be considered acceptable: In three publications, none of the reported patients died during the follow-up period, which ranged from 11 to 156 months after resection [5,6,8]. Dousset et al. and Marangoni et al. [3,7] reported on mortality rates of 14.29% and 27.27% over the follow-up period (up to 51 months and 144 months), respectively. Catalano et al. reported on higher [1] perioperative mortality in the early period < 3 months post-LT compared to >3 months (66.6% vs. 22.2%), with an overall mortality rate of 58.33% of all patients (n = 12) in their cohort in a follow-up of up to 32 months after resection. Overall, the mortality rate during the follow-up period in our center was 57.14%.

The selection of the cases and indications and alternative options massively influence the differing outcomes. More importantly, the time of performing the resection must be well chosen. In cases where perioperative outcome was fatal, presumably choosing to perform the resection at an earlier point in time, “no” resection and/or ReLT, would have been the better choice.

With respect to HCC (either recurrent or de novo), five patients were resected for HCC from the transplanted liver. Four of these patients died within the first two years due to recurrence. Although mortality for this indication was high, surgical treatment is described to be the treatment modality with the most promising effect on a prolonged survival in patients with HCC after LT [11,12,14,15,16]. On the other hand, there is little evidence in liver transplanted patients that reveal similar survival/disease-free survival in patients that receive relapse resection or radiofrequency ablation [10]. Unfortunately, this study does not distinguish between different recurrence sites.

Higher rates of surgical complications in early resections after LT were observed by Catalano et al. [1] and were also seen by Guckelberger et al., who analyzed a cohort of patients undergoing liver resection after LT in our department between August 1988–August 2004 [5]. Marangoni et al. [7] observed the usage of prolonged intensive care units and the hospital stays in patients with early resections compared to late resections. It is noteworthy that Catalano et al. and Marangoni et al. reported only on 3 and 2 patients with liver resection in the early period after LT, respectively.

Four out of fourteen patients (28.6%) were liver resected for localized biliary strictures 13 to 159 months after LT. Those patients survived more than three years after resection, with three of them still alive at the end of the follow-up. The fourth patient died 41 months after resection (for biliary strictures and abscesses) due to a port catheter infection and sepsis. We, therefore, demonstrate that surgical treatment of biliary strictures is a valuable rescue treatment if repeated endoscopic procedures fail to clear biliary infections or to provide a sustainable biliary drainage [1,3].

However, even if the graft is not saved over a long period of time, liver resection can still be an effective option to support the patient until, or to a postponed ReLT. This was the case in 5 out of 66 cases described in the literature. Marangoni et al. reported on a patient who was retranslated due to chronic rejection four months after liver resection for treatment of an infected hematoma of the left liver lobe [7]. In Guckelberger et al. [5], one patient was retransplanted because of IBL 13 months after resection. In the series described by Honoré et al. [6] and Dousset et al. [3], ITBL and recurrent Budd-Chiari Syndrome led to ReLT several months after liver resection.

According to the data from our institution, liver resection after LT is a safe procedure. Nevertheless, it is important to note that three patients did not survive the postoperative hospital stay, which is 21.4%. Obviously, this is inferior to operative mortality in non-transplanted patients, which is described as less than 5% in high volume centers in the literature [17]. Reasons for lower survival in the transplanted cohort is owed to a higher difficulty of the surgical procedure due to previous surgeries, as well as a more complicated clinical management of transplanted patients and worse general medical conditions of those patients. Those are, among other things, reasons why resections in transplanted and non-transplanted patients are not easily comparable.

Still, an alternative to liver resection in most cases remains ReLT. Therefore, the outcome of resection after LT and ReLT was compared. ReLT survival rates in our center (three-year: 55.4%) were comparable with survival rates described in the literature e.g., by Hong et al. who showed overall three-year ReLT survival at 54% [28].

In comparison with the outcome of ReLTs we show the high potential of liver resections in the liver graft compared with ReLT. Liver resections result in similar patient survival, significantly shorter duration of hospital and ICU stay and simultaneously save scarce organs for LT for other patients in need. This should be considered when evaluating treatment options for patients in poor conditions. Additionally, it should be stated that in conditions in which LT is contraindicated, such as in a recurrence of HCC, liver resection in the liver transplant can be the only potentially curative treatment option. For those patients, multiple resections of the same graft seem feasible, as with two patients with HCC recurrence who received two and three liver resections in the same graft. Both patients were alive and tumor-free at the end of the follow-up period.

It is important to note that there are several limitations to this study. First, the overall number of patients with liver resection after LT included in this study is small; however, it still remains, to the best of our knowledge, the largest cohort published to the present day. Moreover, this is a single-center retrospective study covering >10 years, with an immanent heterogeneity regarding patient characteristics, surgical and medical management, as well as quality of the grafts. Another limitation of this study is the choice of the control group. To evaluate the outcome in the resected patients, we chose patients with ReLT as a criterion. This seems reasonable, as ReLT represents a possible treatment alternative, but on the other side, these two patient groups (liver resection and ReLT) differ massively. This is underlined by the fact that propensity score matching was not possible, as the respective e.g., MELD score differed too greatly between these two groups. Therefore, we tried to modify our control group and set exclusion criteria to improve the comparability.

Patients who underwent ReLT in our transplantation center had higher MELD scores than those who were liver resected. Therefore, the effect of shorter hospital stay may also be attributed to lower MELD scores, as these are known risk factors in the course of LT [29]. On the other hand, liver resection enables earlier treatment in the course of the disease with a subsequent better outcome.

Due to the fact that liver resection after LT is only eligible for local pathologies, only a selected number of patients is suitable for this procedure. Most likely, liver resection will not be a standard procedure in the future, but it can be graft-saving and prevented ReLT in all patients in our study cohort, except one.

## 5. Conclusions

Compared to ReLT, resection is a safe procedure, especially in those patients for which ReLT was a potential treatment option at the time of resection. Beneficial aspects of resections after LT are acceptable survival rates and shorter hospital/ICU stays when compared to these aspects from ReLT and saving scarce liver grafts. This is highly relevant not only for patients (eligible or not for retransplantation), but also for health systems facing large discrepancies between patients on the waiting list for LT and liver grafts.

## Figures and Tables

**Table 1:** Literature Review.

Authors	Year	Patients	Indications for LR	Resection Type	Time after LT (Range)	Survival	Outcome	Key Findings
Dousett et al. [3]	1994	7	4 Septic parenchymal infarcts, 3 Nonanastomotic biliary strictures	4 Left Hepatectomies, 2 Left Lobectomies, 1 Right Hepatectomy	3–218 w	1 dead (tumor recurrence); 6 alive (last follow-up 12–45 mo)	2 recurrent ischemic cholangitis, 1 ReLT (recurrent Budd-Chiari Syndrome)	In selected patients with localized ischemic damage to the graft LR is a safe procedure.
Chari et al. [2]	1996	1	1 Ischemic right hepatic lobe	1 Right Hepatic Lobectomy	14 mo	Not mentioned	1 uncomplicated	Due to the preserved regenerative capacity in liver grafts hepatectomies are feasible.
Guerra et al. [9]	1996	2	1 Arterial thrombosis, 1 Biloma and bile duct necrosis	2 Left Lateral Segmentectomy	6 w, 42 mo	----	2 uncomplicated	LR after LT in selected cases is a graft-saving procedure with low morbidity.
Regalia et al. [12]	1998	2	2 HCC recurrence	----	----	2 alive, no recurrence (last follow-up 15 and 67 mo)	----	Surgical resection of HCC recurrence is effective in controlling tumor progression, especially if recurrence appears only in the graft.
Schlitt et al. [14]	1999	3	3 HCC recurrence	----	----	----	----	Surgical resection was the only treatment option compared to chemotherapy and radiotherapy with prolonged survival, especially after late recurrence after LT and should therefore be considered whenever possible.
Honore et al. [6]	2001	4	3 Biliary strictures, 1 Ischemic necrosis	4 Right Hepatic Lobectomies (Seg V, VI, VII, VIII)	2 d–78 mo	4 alive (last follow-up 18–53 mo)	3 uncomplicated, 1 ReLT	Outcome after right hepatic lobectomies in liver grafts is comparable with non-transplanted patients in selected cases.
Catalano et al. [1]	2004	12	5 ITBL, 2 HCC recurrence, 2 Accessory left HAT, 1 Segmental left HAT, 1 Trauma, 1 Liver abscesses	4 Left Lobectomies (Seg II, III); 3 Right Hepatectomies (V, VI, VII, VIII); 2 Extended Right Hemihepatectomies (IV, V, VI, VII, VIII), 1 Right Lateral Sectoriectomy (VI, VII), 1 Anterior Trisegmentomy (IV, V, VI), 1 Segmentectomy (IV)	5 d–1421 d	7 dead (3d–29 mo); 5 alive (last follow-up 3-32 mo)	Early resections (<3 mo after LT): 66.6% (n = 2) perioperative mortality; 1 alive Late resections (>3 months after LT): perioperative mortality 22.2% (n = 2); 2 died of recurrent HCC, 1 died of recurrent HCV, 4 alive	Early diagnosis and timing for LR are crucial, in early LR with sepsis ReLT is preferred. In late LR, timing is important to avoid e.g., the development of sepsis.
Roayaie et al. [13]	2004	5	5 HCC recurrence	----	----	----	----	Patients with tumor recurrence should be resected or ablated. However, it is not clear whether surgical resection or the fact that patients with a tumor which is amendable for resection in a potentially more favorable group, causes higher survival rates.
Guckelberger et al. [5]	2005	3	3 ITBL	3 Left Hepatectomy	13–149 mo	3 alive (last follow-up 12–17 mo)	1 ReLT (13 mo after resection; IBL)	In selected cases LR may prolong the survival of patients without the need of ReLT; however, early LR should be considered with caution.
Marangoni et al. [7]	2008	11	4 HCC recurrence, 2 left HAT, 2 ITBL, 1 Liver Abscesses, 1 Sepsis and infected hematoma, 1 Ischemic segment IV	2 Right Hepatectomies, 1 Extended Right Hepatectomy, 1 Left Hepatectomy, 3 Non-Anatomical Resections, 3 Left Lobectomies, 1 Segmentectomy	0.1–194 mo	3 dead (2 HCC recurrence, 1 PTLD); 8 alive (last follow-up 3–144 mo)	1 ReLT (chronic rejection)	LR are safe and salvage grafts, particularly when performed for ischemic causes. Late resections show shorter ICU and hospital stays. Cure after recurrent HCC is uncommon; however, LR may be beneficial for those perspective patients. In selected patients LR after LT is a safe procedure.
Kornberg et al. [11]	2010	2	2 HCC recurrence	----	----	----	----	If possible, surgical resection should be performed, as it has been shown to be the strongest independent predictor for long-term survival.
Taketomi et al. [15]	2010	4	4 HCC recurrence	----	----	----	----	If available LR may be beneficial for the outcome of recurrence of HCC after LT.
Valdivieso et al. [16]	2010	2	2 HCC recurrence	----	----	----	-----	In patients with resectable HCC recurrence LR should be performed. Although in the whole study cohort (hepatic and/or extrahepatic HCC recurrence) 64% of the patients showed HCC re-recurrence).
Sommacale et al. [8]	2013	8	3 HCC recurrence, 1 Left HAT, 1 Biliary leak, 1 Biliary stricture, 1 Biliary cyst, 1 Hydatid cyst	4 Left lobectomies, 1 Right Hepatectomy, 1 Bisegmentectomy (VI, VII), 1 Biliary Fenestration, 1 Biliary Pericystectomy	5–47 mo	8 alive (last follow-up 11–156 mo)	----	LR is a safe procedure with high morbidity. LR can prevent patients from ReLT, particularly those with resectable HCC.
Huang et al. [10]	2015	11	11 HCC recurrence	----	----	----	----	RFA is a treatment option in recurrent HCC, if surgical options are not applicable with comparable survival.
Pohl et al.	2020	14	5 HCC, 5 Biliary tree pathologies, 2 Bilioma/abscesses, 1 PTLD, 1 Benign tumor	6 Left Hemihepatectomies, 4 Multisegmentectomies, 2 right Hemihepatectomies, 2 Segmentectomies	13–348 mo	8 dead, 6 alive (last follow-up	1 ReLT (abscesses; unsuccessful),	LR after LT is a safe and graft-saving procedure, especially in patients who are potentially eligible for ReLT.

Abbreviations: d, days; w, weeks; mo, months; HAT, Hepatic Artery Thrombosis; HCC, Hepatocellular Carcinoma; HCV, Hepatitis C Virus; IBL, Ischemic Biliary Lesions; ITBL, Ischemic Type Biliary Lesions; LR, Liver Resection; LT, Liver Transplantation; PSC, Primary Sclerosing Cholangitis; PTLD, Post-Transplant Lymphoproliferative Disease; ReLT, Retransplantation; RFA, Radiofrequency ablation.

**Table 2:** Individual Characteristics of Patients Undergoing Liver Resection after Liver Transplantation.

Patient	Sex	Age at Resection [Years]	Indication for LT	Indication for Liver Resection	Type of Resection	Months after LT	MELD at Resection	Retransplantation Possible	Clavien/ Dindo	Immunosuppressive Regimen	Comorbidities	Follow Up (after Resection)	Status	Cause of Death
No. 1	m	64	Alcoholic cirrhosis	HCC	Segmentectomy (IV, V, VIII)	194	21	yes	II	Tacrolimus, MMF	Coronary heart disease; ESRD; HT; COPD; DM II	7 mo	Death	HCC
No. 2	m	58	HCC	HCC	Left hemihepatectomy	14	-	no	IV	Tacrolimus	HT; DM II	6 mo	Death	HCC Recurrence
No. 3	m	29	AIH	Localized biliary stricture	Left hemihepatectomy & extrahepatic bile duct resection	159	14	yes	III	Tacrolimus, Prednisolone	-	52 mo	Alive	
No. 4	m	52	HCC	Localized biliary stricture	Atypical segmentectomy (V, VIII)	13	7	yes	III	Tacrolimus	DM II; COPD	41 mo	Death	Sepsis
No. 5	f	58	HCV Cirrhosis	HCC	Segmentectomy (IV) ;(Segmentectomy (II), 09/2018; left hemihepatectomy, 02/2019)	79	6	yes	-	Tacrolimus, Everolimus	Chronic Hepatitis C	30 mo	Alive	
No. 6	f	66	PBC	Localized biliary stricture	Left hemihepa tectomy & segmentectomy (I), extrahepatic bile duct resection	130	6	yes	II	Tacrolimus	-	49 mo	Alive	
No. 7	m	64	Alcoholic cirrhosis	Liver abscess	Right hemihepatectomy and segmentectomy (I)	271	15	yes	V	Tacrolimus, MMF	Coronary heart disease; Depression; DM II; HT; COPD	2 d	Death	Graft Dysfunction
No. 8	m	52	PSC	PSC	Left hemihepatectomy	60	21	yes	III	Tacrolimus	Coronary heart disease; Dilatative cardiomyopathy; Tricuspid and mitral insufficiency; Sjögren syndrome	20 mo	Death	Cerebrovascular accident
No. 9	m	65	HCC	Localized biliary stricture	Left hemihepatectomy	82	32	yes	IV	Tacrolimus, MMF	-	67 mo	Alive	
No. 10	f	43	Cryptogenic cirrhosis	PTLD	Right hemihepatectomy	166	8	no	-	Tacrolimus	-	84 mo	Alive	
No. 11	f	64	HCC	Liver abscesses	Left hemihepatectomy	348	8	no	V	Azathioprine, Prednisolone	Paralytic ileus; Sepsis; Scoliosis; Osteogenesis imperfecta; Aneurysms A. phrenica dextra and A. hepatis communis	39 d	Death	Sepsis
No. 12	m	60	HCC	Suspected HCC; (HCC 03/2001)	Atypical segmentectomy (V, VI); (Segmentectomy (II, III), 03/2001)	111	-	yes	-	Sirolimus,	Chronic Hepatitis B	158 mo	Alive	
No. 13	m	69	HCC	HCC	Segmentectomy (II, III)	46	-	no	V	Tacrolimus	Paroxysmal atrial fibrillation	46 d	Death	Hemorrhagic shock after hepatic tumor rupture
No. 14	m	65	HBV cirrhosis	HCC	Segmentectomy (VI)	215	-	no	-	MMF	Hepatitis B reinfection after LT; ESRD; HT; COPD; Coronray heart disease	19 months	Death	HCC

Abbreviations: d, days; f, female; m, male; mo, months; AIH, Autoimmune Hepatitis; COPD, Chronic Obstructive Pulmonary Disease; DM II, Diabetes mellitus Type 2; ESRD, End-stage Renal Disease; HCC, Hepatocellular Carcinoma; HT, Arterial Hypertension; ICU, Intensive Care Unit; LT, Liver transplantation; MMF, mycophenolate mofetil; PBC, Primary Biliary Cholangitis; PSC, Primary Sclerosing Cholangitis; PTLD, Post-Transplant Lymphoproliferative Disorder; ReLT, Liver Retransplantation.

**Table 3:** Characteristics of the study cohort.

	LR after LT	LR after LT (ReLT Possible)	ReLT after LT (All Patients)	vs. LR	ReLT after LT (Late ReLT)	vs. LR
Patients, total *	14	9	175		44	
Gender [male/female] *	10/4	7/2	96/79		25/19	
Age [years] **	57.8 (±10.9)	56.67 (±11.65)	49.0 (±10.7)	***p* = 0.003**	47.55 (±11.76)	***p* = 0.003**
Hospital Stay [days] ***	25 (8.75–49)	17 (7.5–52)	44 (25–75)	***p* = 0.017**	40 (24–68.25)	***p* = 0.034**
ICU Stay [days] ***	2 (1–8)	2 (1–5)	19 (8–49)	***p* < 0.001**	16 (8–29.75)	***p* < 0.001**
Indication for LT *			Indication for ReLT *			
HCC	6 (42.9%)	3 (33.3%)	Primary non-function	41 (25.4%)	0 (0.0%)	
Cirrhosis (alcohol/nutritive/viral)	4 (28.6%)	3 (33.3%)	Hepatic artery thrombosis	37 (21.1%)	5 (11.4%)	
Cryptogenic Cirrhosis/AIH	2 (14.3%)	1 (11.1%)	ITBL	28 (16.0%)	15 (34.1%)	
PBC/PSC	2 (14.3%)	2 (22.2%)	Recurrent disease after LT	20 (11.4%)	14 (31.8%)	
			Rejection	14 (8.0%)	0 (0.0%)	
			Others, not specified	35 (20.0%)	10 (22.7%)	
Indication for Resection *						
HCC	5 (35.7%)	2 (22.2%)				
Biliary tree pathologies	5 (35.7%)	5 (55.6)				
Abscesses and bilioma	2 (14.3%)	1 (11.1%)				
PTLD	1 (7.1%)	-				
Benign tumor	1 (7.1%)	1 (11.1%)				
Days after Liver Transplantation ***	3682.5 (1725–6073.5)	3401.0 (2116.5–5383.5)	19.5 (6–2215.75)	***p* < 0.001**	3523.5 (1737–5015.5)	*p* = 0.778
MELD at time of resection/retransplantation ***	11 (6.75–21)	14.5 (6.25–21)	31 (21–36)	***p* < 0.001**	32 (17.5–37)	***p* = 0.001**
Survival						
three-year Survival	50.0%	66.7%	55.4%	*p* = 0.913 (vs. LR ReLT possible: *p* = 0.448)	59.1%	*p* = 0.733 (vs. LR ReLT possible: *p* = 0.598)

* Frequency/Quantity; ** Mean (± standard deviation); *** Median (interquartile range); Statistically significant p-values are in bold; Abbreviations: LR, Liver Resection; LT, Liver transplantation; ReLT, Liver Retransplantation; ICU, Intensive Care Unit; HCC, Hepatocellular Carcinoma; AIH, Autoimmune Hepatitis; PBC, Primary Biliary Cholangitis; PSC, Primary Sclerosing Cholangitis; ITBL, Ischemic Type Biliary Lesions; PTLD, Post-Transplant Lymphoproliferative Disorder.

## Abbreviations

HCC	Hepatocellular carcinoma
HCV	Hepatitis C virus
IBL	Ischemic Biliary Lesions
ITBL	Ischemic Type Biliary Lesions
IQR	Interquartile Range
LT	Liver Transplantation
MELD	Model for End-Stage Liver Disease
POD	Postoperative Day
ReLT	Liver Retransplantation
SD	Standard Deviation
